# Paeonol regulates NLRP3 inflammasomes and pyroptosis to alleviate spinal cord injury of rat

**DOI:** 10.1186/s12868-022-00698-9

**Published:** 2022-03-18

**Authors:** Houling Zhao, Xi Wang, Shuheng Liu, Qingguo Zhang

**Affiliations:** 1 Department of Orthopaedic Trauma, Central Hospital Affiliated to Shandong First Medical University, No. 105, Jiefang Road, Jinan City, 250000 Shandong Province China; 2 Department of Spinal Surgery, Central Hospital Affiliated to Shandong First Medical University, No. 105, Jiefang Road, Jinan City, 250000 Shandong Province China

**Keywords:** Paeonol, Spinal cord injury, NLRP3 inflammasomes, Pyroptosis, TLR4/MyD88/NF-κB

## Abstract

**Background:**

Spinal cord injury (SCI) is a life-threatening traumatic disorder. Paeonol has been confirmed to be involved in a variety of diseases. The purpose of this study is to investigate the role of paeonol on SCI progression.

**Methods:**

Sprague Dawley (SD) rat was used for the establishment of SCI model to explore the anti-inflammation, anti-oxidation, and neuroprotective effects of paeonol (60 mg/kg) on SCI in vivo. For in vitro study, mouse primary microglial cells (BV-2) were induced by lipopolysaccharide (LPS)/adenosine triphosphate (ATP) treatment. The effect of paeonol on the polarization of LPS/ATP-induced BV-2 cells was determined by detection the expression inducible nitric oxide synthase (iNOS), tumour necrosis factor alpha (TNF-α), arginase-1 (Arg-1), and interleukin (IL)-10 using qRT-PCR. ELISA was used to assess the levels of IL-1β, IL-18, TNF-α, malondialdehyde (MDA), and glutathione (GSH). Western blotting was conducted to determine the levels of NLRP3 inflammasomes and TLR4/MyD88/NF-κB (p65) pathway proteins.

**Results:**

Paeonol promoted the recovery of locomotion function and spinal cord structure, and decreased spinal cord water content in rats following SCI. Meanwhile, paeonol reduced the levels of apoptosis-associated speck-like protein (ASC), NLRP3, active caspase 1 and N-gasdermin D (N-GSDMD), repressed the contents of IL-1β, IL-18, TNF-α and MDA, and elevated GSH level. In vitro, paeonol exerted similarly inhibiting effects on pyroptosis and inflammation. Meanwhile, paeonol promoted BV-2 cells M2 polarization. In addition, paeonol also inactivated the expression of TLR4/MyD88/NF-κB (p65) pathway.

**Conclusion:**

Paeonol may regulate NLRP3 inflammasomes and pyroptosis to alleviate SCI, pointing out the potential for treating SCI in clinic.

## Introduction

Spinal cord injury (SCI) is a severe and disabling trauma disease, which is mainly caused by traffic accidents and high-altitude falling [1]. Approximately 250,000 to 500,000 individuals suffer from the pain of SCI annually worldwide [2, 3]. SCI generally results in neurological dysfunction, reduces the quality of life, or even threatens patient’s life [4, 5]. Despite the increasing of treatment costs each year, there is still no effective therapy to enhance neurological recovery after SCI [6]. Therefore, exploring an effective clinical-drug is urgent to alleviate SCI.

Neuroinflammation is widely known as an immune response in the central nervous system (CNS), which plays an important role in the functional recovery of nervous tissues after SCI [7, 8]. Increasing attention has been paid to the functions of inflammasomes, especially NLRP3 inflammasomes, a kind of subcellular multiprotein complexes [9]. In general, NLRP3 inflammasomes are highly expressed in CNS to detect the invading agents [10]. However, the activation of NLRP3 inflammasomes further activated the expression of caspase 1, apoptosis-associated speck-like protein (ASC) and N-gasdermin D (N-GSDMD), which is accompanied by the production of interleukin (IL)-18 and IL-1β [11–13]. In addition, numerous studies have uncovered that the formation of inflammasomes is strongly correlated with pyroptosis, eventually contributing to the secretion of inflammatory cytokines [14–18]. In the process of pyroptosis, caspase-1 may interact with ASC to form inflammasomes, while inflammasomes further actives caspase-1 to form active caspase 1. The active caspase 1 further promotes the activation of GSDMD to form N-GSDMD and C-GSDMD, while N-GSDMD eventually results in pyroptosis [16]. Meanwhile, a large number of IL-18 and IL-β was released from the impaired microglial cells [17, 18]. Hence, controlling the activation of NLRP3 inflammasomes and inhibiting pyroptosis of microglial cells may be helpful to attenuate SCI.

Paeonol (2’-hydroxy-4’-methoxyacetophenone) is the main active component in the extract of peony root [19]. Report on the clinical application of paeonol can be traced back to 1985 [20]. With the development of medical technology, growing clinical applications of paeonol have been uncovered, such as the inhibitory role in inflammation [21, 22], cardiovascular diseases [23, 24], tumor [25, 26], and oxidative stress [27, 28]. For instance, Zhai et al. have indicated that paeonol can attenuate rheumatoid arthritis through mediating NF-κB signaling pathway [21]. Paeonol inactivates TLR4 signaling pathway to repress the apoptosis of lipopolysaccharide (LPS)-treated endothelial cells [23], and inhibits NF-κB signaling pathway to accelerate the apoptosis of gastric cancer cells [25].Additionally, paeonol can relieve the hepatotoxicity via increasing glutathione (GSH) level and decreasing malondialdehyde (MDA) content [27]. Notably, the neuroprotective roles of paeonol on numerous central nervous system disorders are also determined, including Alzheimer's disease [29], cerebral ischemic injury [30, 31], Parkinson's disease [32], and diabetic encephalopathy [33]. SCI, as a well-known neurological dysfunction disorder accompanied by the occurrence of inflammation and oxidative stress [34, 35], there are still no relevant researches concentrated on the function of paeonol in SCI progression. More importantly, a recent study has reported that paeonol may attenuate NLRP3 mediated inflammation in a hyperlipidemia rat model [36]. However, the action mechanism of paeonol on NLRP3 inflammasomes, and the interactions between paeonol and pyroptosis in SCI are still unclear.

In this study, the regulatory mechanisms of paeonol, and the interactions among paeonol, NLRP3 inflammasomes, and pyroptosis in SCI (in vitro and in vivo models) were preliminarily investigated. Our findings indicate that paeonol may serve as a potential therapeutic agent for treating SCI.

## Results

### Paeonol attenuates SCI in a rat model

**Fig. 1:**
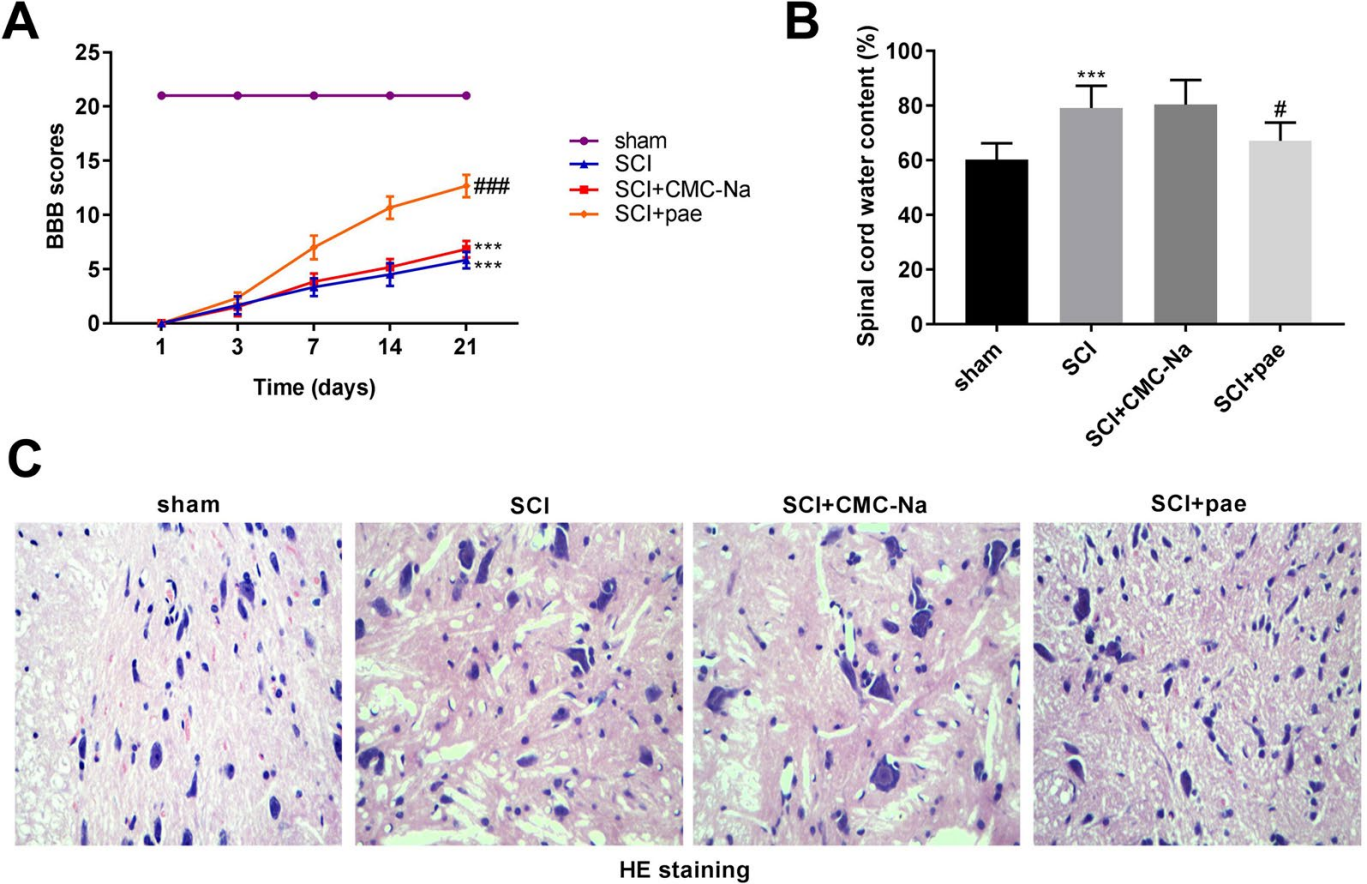
Paeonol attenuates SCI in a rat model. **A** The BBB scores at 1, 3, 7, 14, and 21 days after SCI. ***P < 0.001 vs. the sham group. ^###^*P* < 0.001 vs. the SCI + CMC-Na group. **B** The spinal cord water content of the sham, SCI, SCI + CMC-Na, and SCI + pae groups. ***P < 0.001 vs. the sham group. ^#^*P* < 0.05 vs. the SCI + CMC-Na group. **C** The statuses of rat spinal cord tissues at 7 days post SCI were determined by H&E staining assay. Magnification × 200

To explore the therapeutic efficacy of paeonol, we first established a rat SCI model. As presented in Fig. 1A, we found that the BBB scores of rats in the SCI group (*P* < 0.001) or SCI + CMC-Na group (*P* < 0.001) were significantly decreased compared to those in the sham group, whereas paeonol treatment had a remarkable improvement on rat SCI (*P* < 0.001). Unsurprisingly, the content of spinal cord water in the SCI group was relatively higher than that of sham rat (Fig. 1B, *P* < 0.001). Spinal cord water content in the SCI + pae group was significantly reduced compared to that in the SCI group (*P* < 0.05). Next, H&E staining was performed to further validate the protective effect of paeonol on SCI. As illustrated in Fig. 1C, we discovered that the structure of spinal cord in the sham group was normal, while the spinal cord structures of the SCI group and SCI + CMC-Na group got damaged with the formation of some cavities. At the same time, the architecture of the spinal cord was better preserved in the SCI + paeonol group.

### Paeonol represses the pyroptosis and formation of NLRP3 inflammasome in SCI rat model

**Fig. 2:**
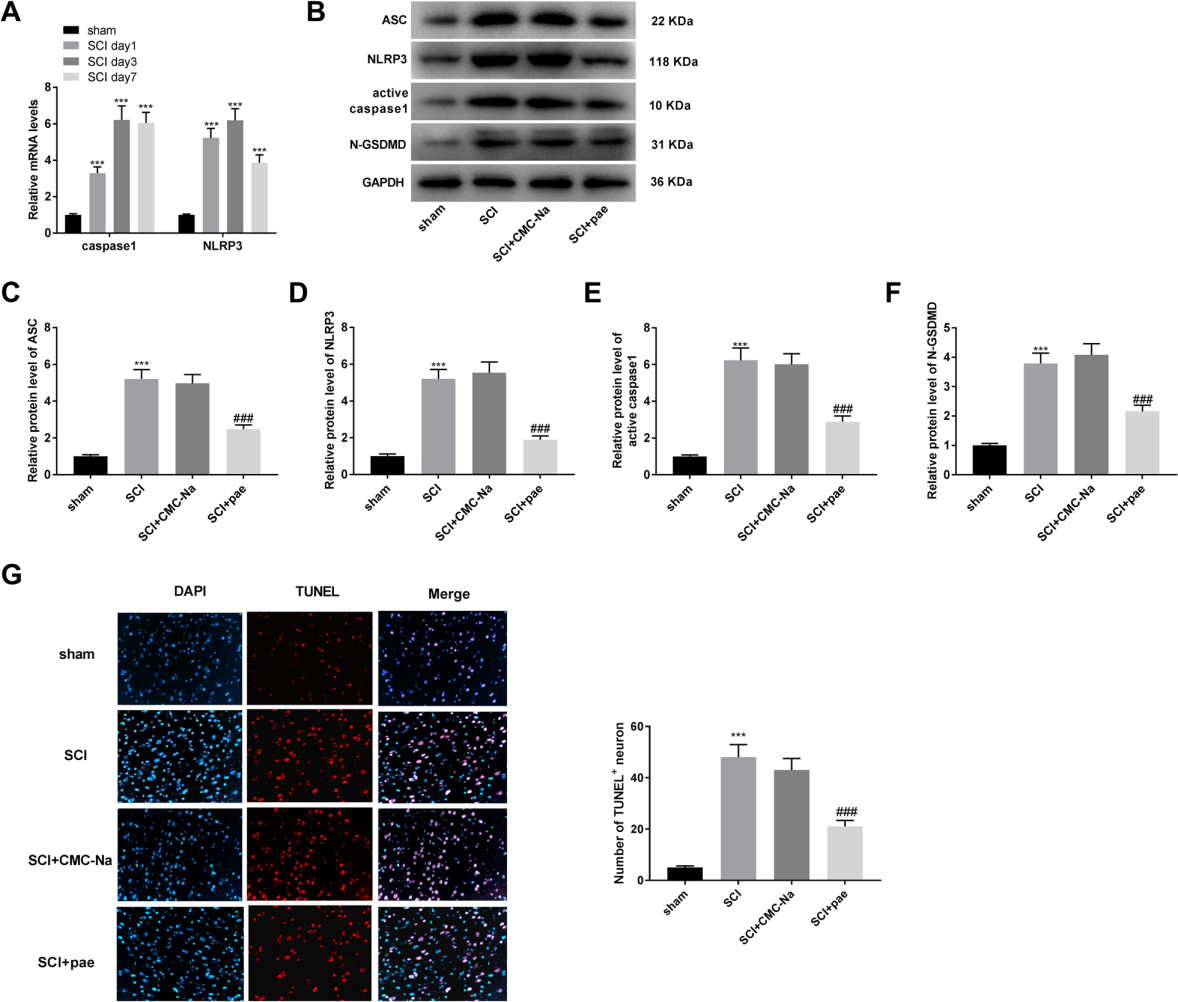
Paeonol represses the pyroptosis and formation of NLRP3 inflammasome in SCI rat model. **A** The mRNA expression of caspase1 and NLRP3 at 1, 3, and 7 days after SCI. ***P < 0.01 vs. the sham group. (B) The western blot assay images (20 μg/lane) for the levels of ASC, NLRP3, active caspase 1, and N-GSDMD in spinal cord tissues. (C) The protein level of ASC in spinal cord tissues was measured by western blot assay. (D) The protein level of NLRP3 in spinal cord tissues was measured by western blot assay. (E) The protein level of active caspase 1 in spinal cord tissues was measured by western blot assay. (F) The protein level of N-GSDMD in spinal cord tissues was measured by western blot assay. (G) The number of TUNEL-positive neuron was measured by TUNEL staining assay. ***P < 0.001 vs. the sham group. ^###^*P* < 0.001 vs. the SCI + CMC-Na group

The mRNA levels of caspase 1 and NLRP3 at different time points after SCI were initially determined. Caspase1 mRNA level in spinal cord tissues of SCI rat was elevated at day 1, peaked at day 3 and persisted at a relatively high level at day 7 (Fig. 2A, *P* < 0.001). Although the mRNA level of NLRP3 at day 7 was relatively lower than that at day 1 or day 3, NLRP3 mRNA concentration in SCI rats at the three time points were all significantly increased compared to the sham rats (*P* < 0.001). Meanwhile, the protein levels of ASC, NLRP3, active caspase 1, and N-GSDMD in spinal cord tissues of rat were measured by western blot assay. The results demonstrated that SCI significantly increased these protein levels (Fig. 2B–F, *P*  < 0.001), whereas these promoting effects caused by SCI were reversed by paeonol treatment (*P* < 0.001). Additionally, based on the results of TUNEL staining assay, SCI group showed increased number of TUNEL-positive neuron, while paeonol administration revised this situation (Fig. 2G, *P*  < 0.001).

### Paeonol alleviates the neuroinflammation and oxidative stress in SCI rat model

**Fig. 3:**
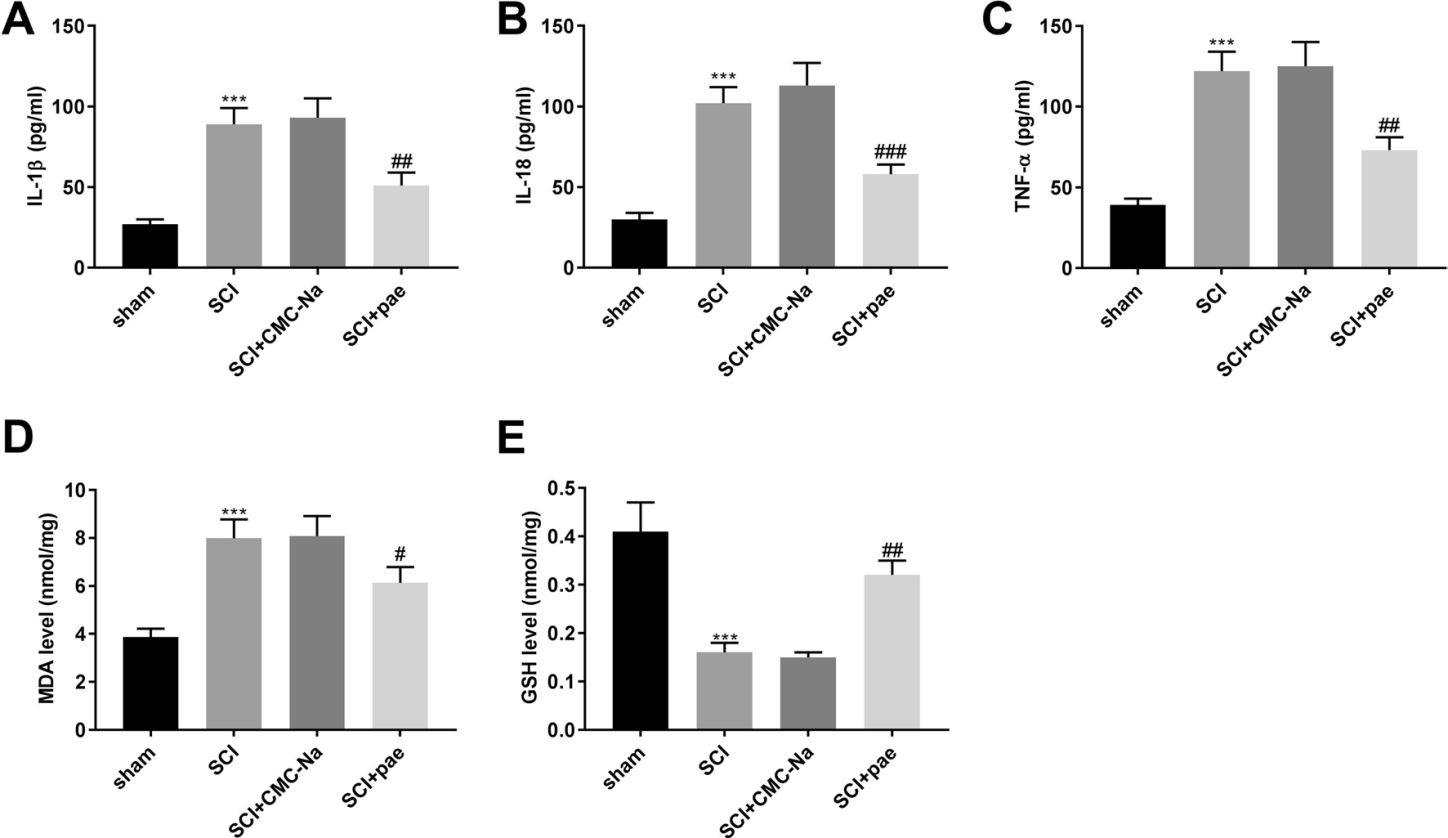
Paeonol alleviates the neuroinflammation and oxidative stress in SCI rat model. **A** The level of IL-1β in spinal cord tissues was measured by ELISA. **B** The level of IL-18 in spinal cord tissues was measured by ELISA. **C** The level of TNF-α in spinal cord tissues was measured by ELISA. **D** The level of MDA in spinal cord tissues was measured by a corresponding commercial assay kit. **E** The level of GSH in spinal cord tissues was measured by a corresponding commercial assay kit. ***P < 0.001 vs. the sham group. ^#^*P* < 0.05, ^##^*P* < 0.01, ^###^*P* < 0.001 vs. the SCI + CMC-Na group

The possible role of paeonol on neuroinflammation and oxidative stress of rat after SCI was further assessed. As shown in Fig. 3A–C, paeonol treatment reversed the increased levels of IL-1β, IL-18, and TNF-α induced by SCI (*P* < 0.01). Additionally, we found a high level of MDA and a low level of GSH in rat spinal cord tissues following SCI (Fig. 3D–E, *P*  < 0.001), while these situations were partly reversed in rats injection of paeonol (*P* < 0.05).

### Effects of paeonol on microglia and astrocyte

**Fig. 4:**
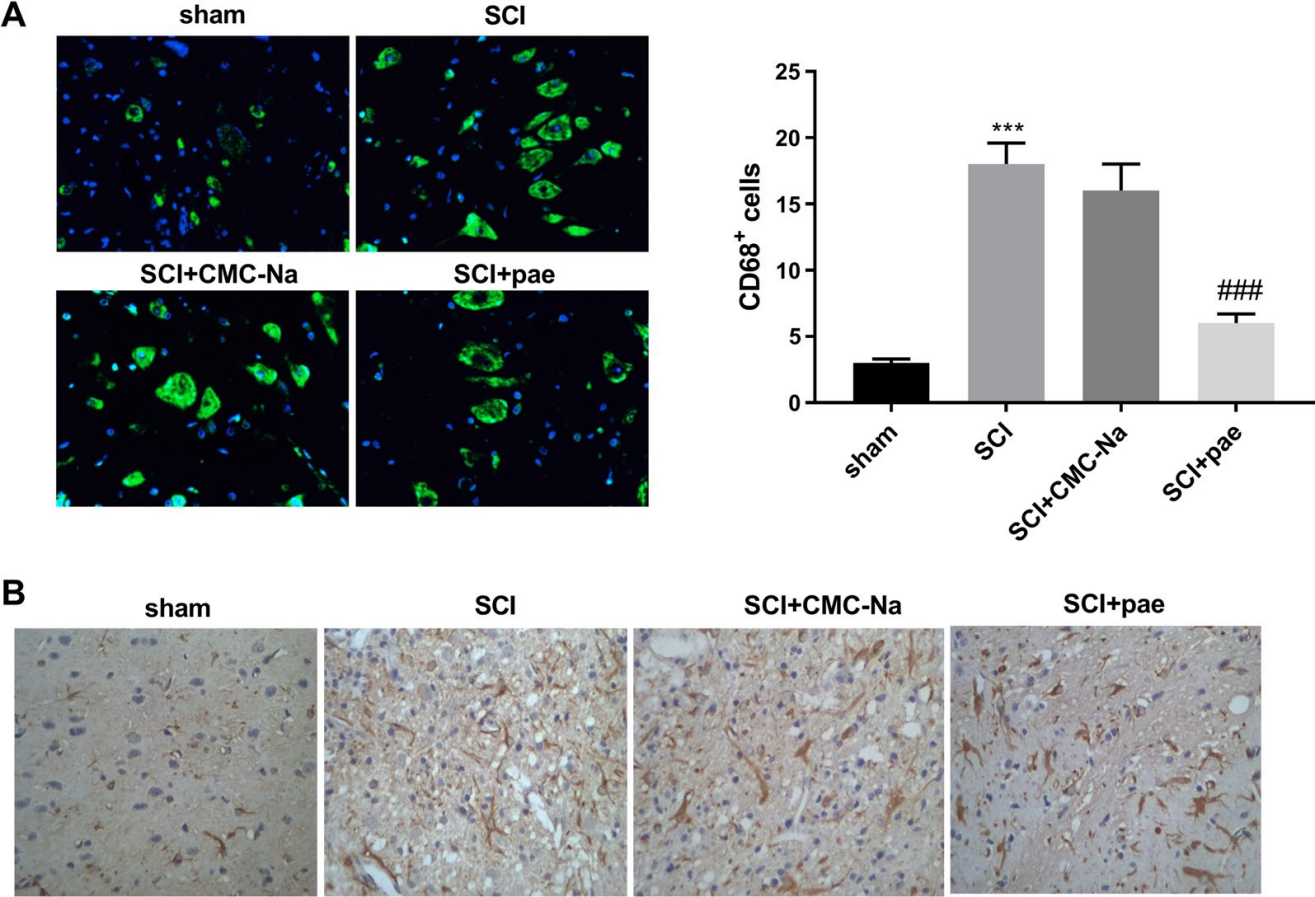
Effects of paeonol on microglia and astrocyte. **A** Immunofluorescence labeling to estimate the number of CD68-positive cells. **B** IHC analysis to assess GFAP immunoreactivity. ***P < 0.001 vs. the sham group. ^###^*P* < 0.001 vs. the SCI + CMC-Na group

To determine the number of activated microglia, CD68 expression was estimated by immunofluorescence labelling. SCI induced a significant increase in the number of activated microglia, as demonstrated by an elevation in CD68-positive cells (Fig. 4A, *P*  < 0.001). Such an alteration was remarkably reversed by paeonol administration (*P* < 0.001). To analyze the reactive astrogliosis that mediated the formation of glial scar, GFAP immunoreactivity was estimated by IHC analysis. As shown in Fig. 4B, GFAP immunoreactivity was robustly elevated after SCI; these changes were markedly normalized by paeonol treatment.

### Paeonol promotes BV-2 cells M2 polarization

**Fig. 5:**
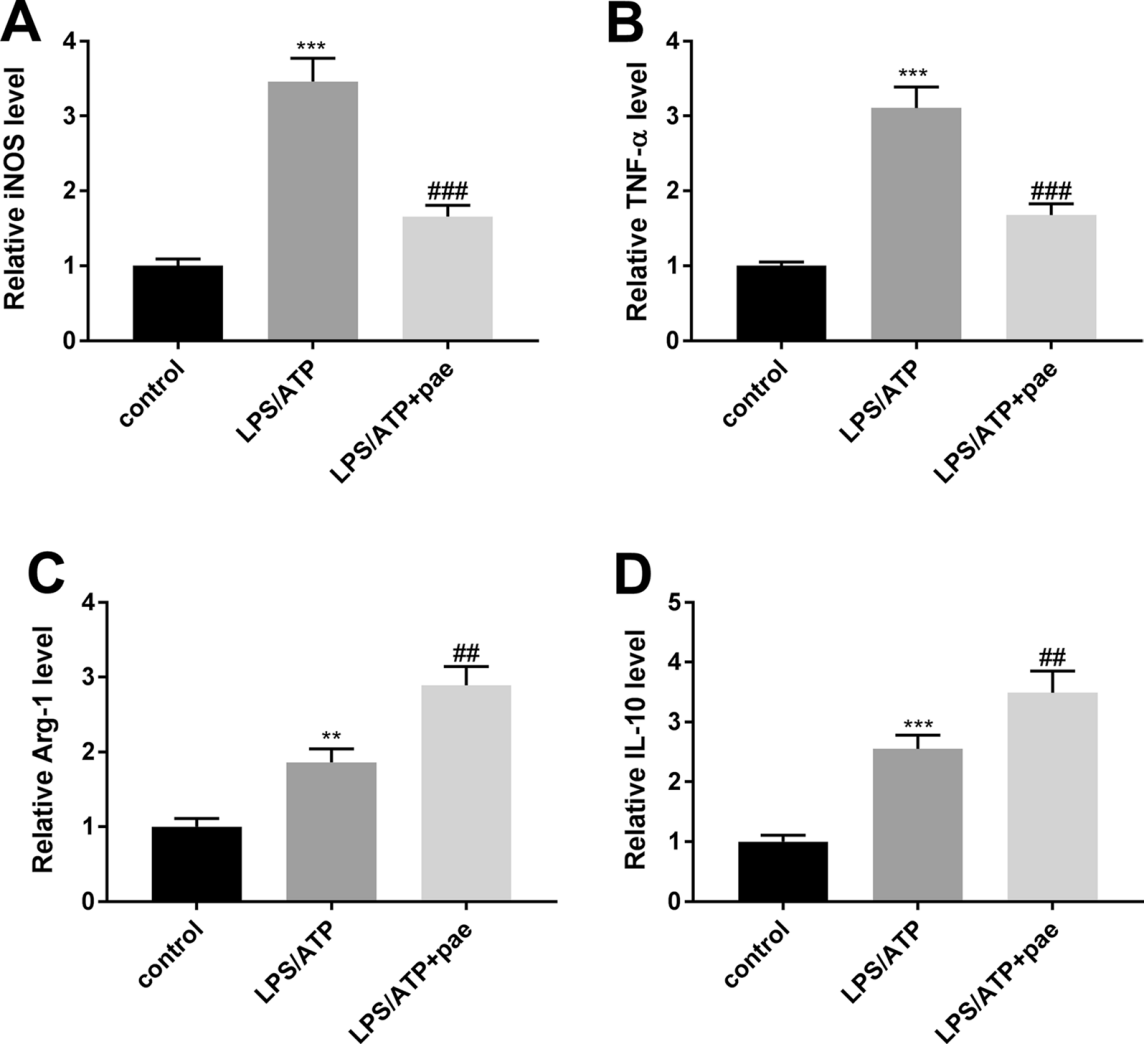
Paeonol promotes BV-2 cells M2 polarization. **A** The expression level of iNOS in BV-2 cells was detected by qRT-PCR. **B** The expression level of TNF-α in BV-2 cells was detected by qRT-PCR. **C** The expression level of Arg-1 in BV-2 cells was detected by qRT-PCR. **D** The expression level of IL-10 in BV-2 cells was detected by qRT-PCR. **P < 0.01, ***P < 0.001 vs. the control group. ^##^*P* < 0.01, ^###^*P* < 0.001 vs. the LPS/ATP group

To explore the function of paeonol on microglia polarization, the mRNA levels of M1 polarization markers (iNOS and TNF-α) and M2 polarization markers (Arg-1 and IL-10) were studied. The mRNA levels of iNOS and TNF-α were remarkably increased in the LPS/ATP group (Fig. 5A, B, *P*  < 0.001), and decreased in the LPS/ATP + paeonol group (*P* < 0.001). However, the levels of Arg-1 and IL-10 showed the opposite pattern (Fig. 5C, D, *P*  < 0.01).

### Paeonol suppresses the pyroptosis and inflammatory responses in BV-2 cells

**Fig. 6:**
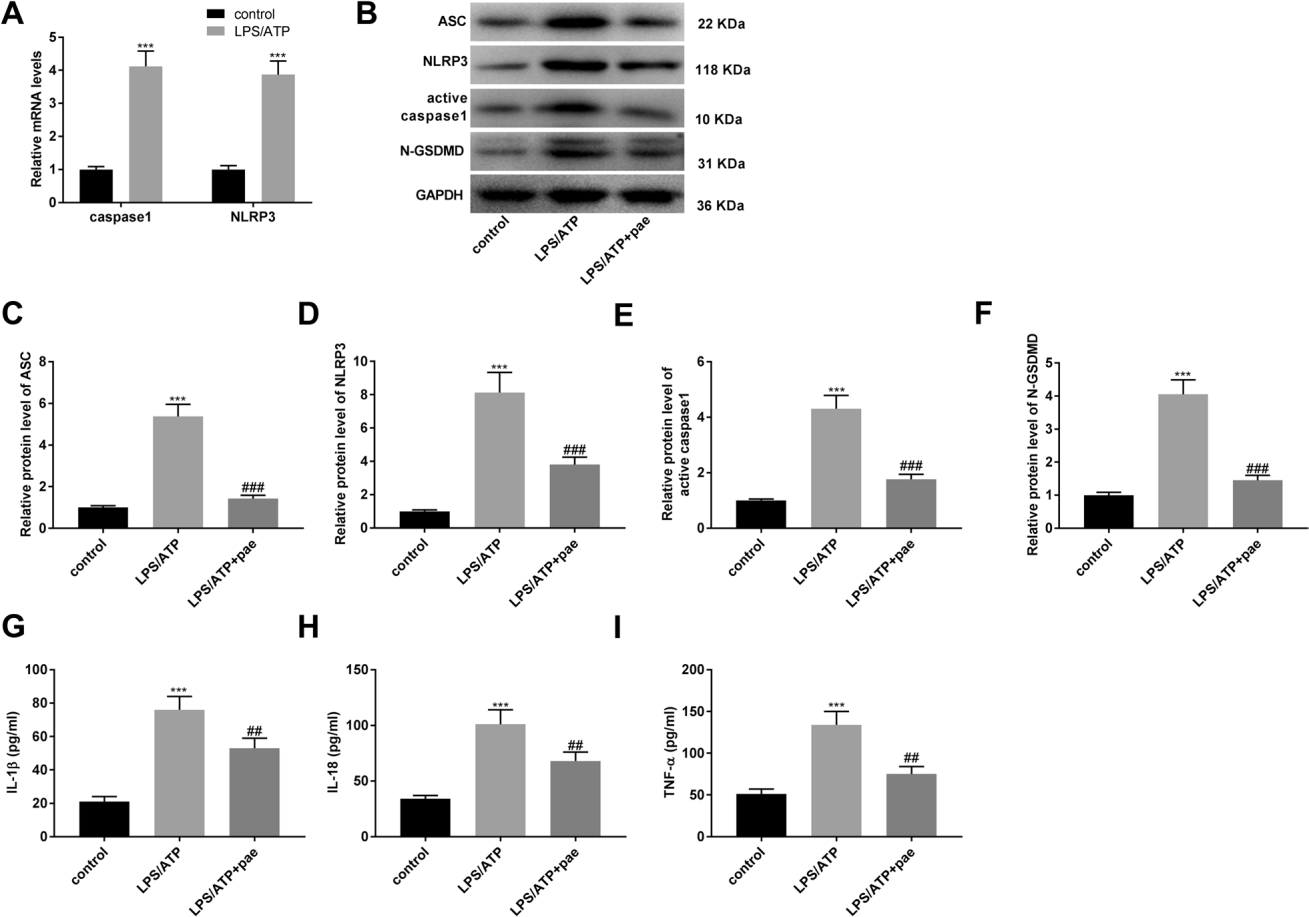
Paeonol suppresses the pyroptosis and inflammatory responses in BV-2 cells. **A** The mRNA expression of caspase1 and NLRP3 in BV-2 cells was detected by qRT-PCR. ***P < 0.001 vs. the control group. **B** The western blot assay images (20 μg/lane) for the levels of ASC, NLRP3, active caspase 1, and N-GSDMD in BV-2 cells. **C** The protein level of ASC in BV-2 cells was measured by western blot assay. **D** The protein level of NLRP3 in BV-2 cells was measured by western blot assay. **E** The protein level of active caspase 1 in BV-2 cells was measured by western blot assay. **F** The protein level of N-GSDMD in BV-2 cells was measured by western blot assay. **G** The level of IL-1β i in BV-2 cells was measured by ELISA. **H** The level of IL-18 in BV-2 cells was measured by ELISA. **I** The level of TNF-α in BV-2 cells was measured by ELISA. ***P < 0.001 vs. the control group. ^##^*P* < 0.01, ^###^*P* < 0.001 vs. the LPS/ATP group

In order to understand the effects of paeonol on pyroptosis and inflammation in BV-2 cells, in vitro experiments were performed. The mRNA levels of caspase 1 and NLRP3 in BV-2 cells were remarkably increased in the LPS/ATP group compared to the control group (Fig. 6A, *P*  < 0.001). As presented in Fig. 6B–F, addition of paeonol reversed the promoting effects of LPS and ATP co-treatment on the protein levels of ASC, NLRP3, active caspase 1, and N-GSDMD (*P* < 0.001). Similarly, the results of ELISA uncovered that the levels of IL-1β, IL-18, and TNF-α were also elevated in the LPS/ATP group (Fig. 6G–I, *P*  < 0.001), while these patterns were reversed in the LPS/ATP + paeonol group (*P* < 0.01).

### Paeonol inactivates the TLR4/MyD88/NF-κB (p65) signalling pathway in BV-2 cells

**Fig. 7:**
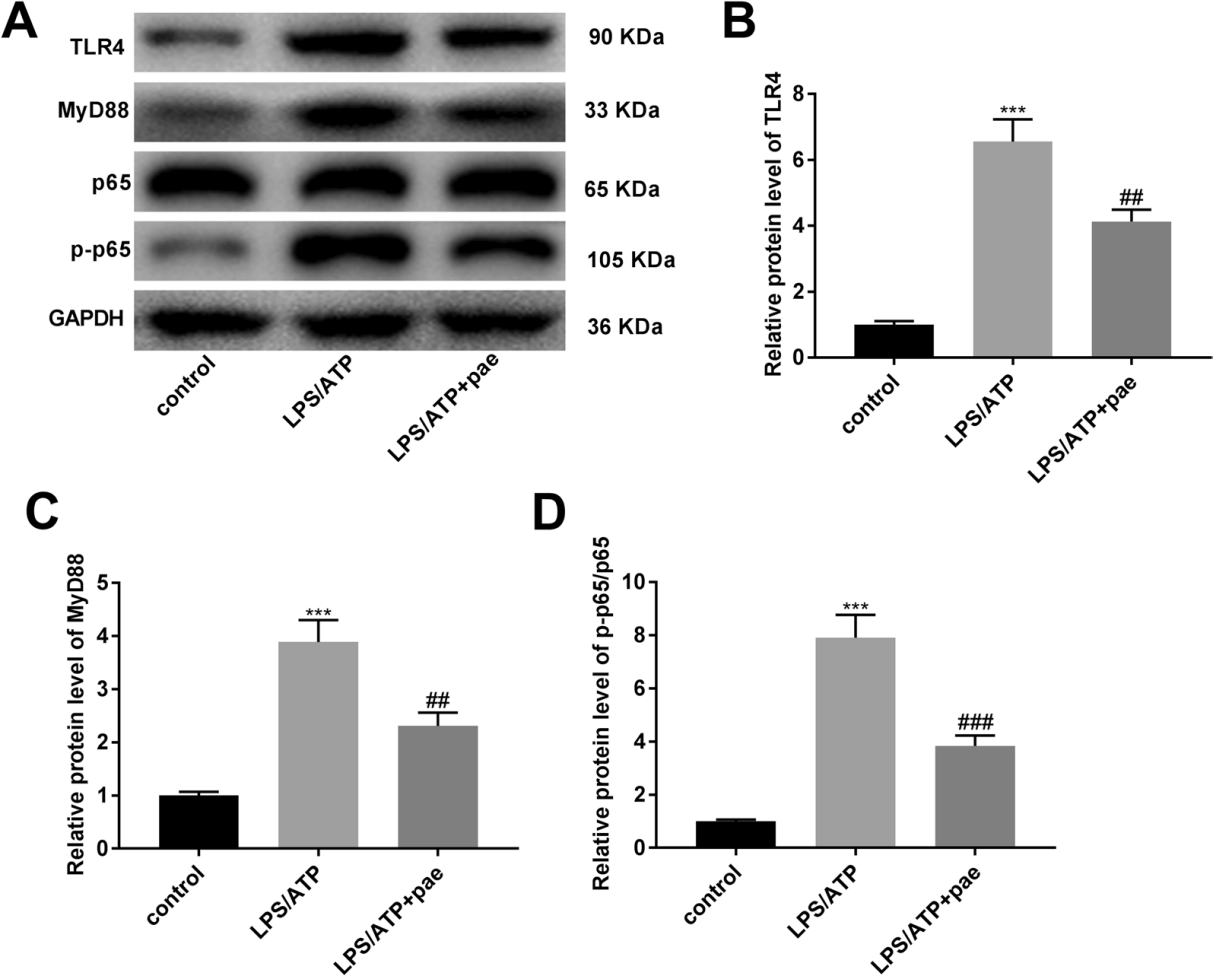
Paeonol makes inactivation for the TLR4/MyD88/NF-κB (p65) signalling pathway in BV-2 cells. **A** The western blot assay images (20 μg/lane) for the levels of TLR4, MyD88, p65, and p-p65 in BV-2 cells. **B** The protein level of TLR4 in BV-2 cells was measured by western blot assay. **C** The protein level of MyD88 in BV-2 cells was measured by western blot assay. **D** The protein level of p-p65/p65 in BV-2 cells was measured by western blot assay. ***P < 0.001 vs. the control group. ^##^*P* < 0.01, ^###^*P* < 0.001 vs. the LPS/ATP group

Because the TLR4/MyD88/NF-κB (p65) signalling pathway is a primary pathway involved in neuroinflammation [37, 38], the protein levels of TLR4, MyD88, and p-p65/p65 were determined for further validation the role of paeonol in LPS/ATP-induced BV-2 cells. As illustrated in Fig. 7A–D, we found that the levels of TLR4, MyD88, and p-p65/p65 were increased in the LPS/ATP group (*P* < 0.001), whereas these situations were reversed in the LPS/ATP + paeonol group (*P* < 0.01).

## Discussion

Inflammatory response is an important factor of secondary injury in SCI [39, 40]. The adoption of various measures to prevent or suppress inflammation has become a means of SCI treatment. In this study, we explore the protective effect of paeonol on the damages to spinal cord structure, and indicate that it may relieve the acute phase of the inflammatory response via inhibiting NLRP3 inflammasomes formation, microglia pyroptosis, and TLR4/MyD88/NF-κB (p65) pathway.

Emerging researches have revealed the neuroprotective effect of paeonol on central nervous system diseases [41, 42]. It is well known that SCI is characterized by the disordered spinal cord structure and cavity formation [43]. In addition, SCI rats generally show impaired locomotion function and increased spinal cord water content [43, 44]. In this study, we discovered that paeonol could effectively reduce spinal cord water content and relieve the damages to spinal cord structure in rats following SCI, suggesting that paeonol may be an underlying agent to attenuate SCI.

NLRP3 inflammasome is considered as an important factor in the progression of SCI [43, 45]. A previous study has revealed that celastrol can protect rat against the SCI through inactivation of NLRP3 inflammasomes [43]. Jiang et al. used the pharmacologic inhibitor BAY 11-7082 or A438079 to specifically repress the activation of NLRP3 inflammasomes and found that inhibition of NLRP3 inflammasomes reduces neuronal death, attenuates spinal cord anatomic damage, decreases the levels of inflammatory cytokines, and promotes motor recovery [45]. These researches implied that NLRP3 inflammasomes are a vital contributor to the secondary damage of SCI. Similarly, paeonol interacts with NLRP3 inflammasomes in a hyperlipidemic rat model has revealed that paeonol can reduce the levels of NLRP3, active caspase 1, and ASC to alleviate rat hyperlipidemia [36]. In the current study, we found that paeonol treatment significantly repressed the levels of NLRP3 inflammasomes-related proteins both in vitro and in vivo. Our data suggested that the interventions of paeonol on SCI may achieve by regulation of NLRP3 inflammasomes.

Pyroptosis is involved in another crucial cellular process and has synergistic effect with NLRP3 inflammasomes in the development of SCI [43, 46]. The activated NLRP3 inflammasomes can further induce the cleavage of GSDMD, eventually triggering pyroptosis [46]. Therefore, targeting pyroptosis and inflammasome components can be novel therapeutic strategies for SCI [47]. In this study, we demonstrated that the levels of pyroptosis-related proteins were decreased by paeonol treatment in rats following SCI and in LPS/ATP-induced microglia, which implied that the participation of pyroptosis in SCI progression can be regulated by paeonol. These results are further confirmed by TUNEL assay. Furthermore, the activation of NLRP3 inflammasomes and pyroptosis is accompanied with the release of inflammatory cytokines [36, 43, 47]. In line with the previous studies, we found that paeonol decreased the high levels of IL-1β, IL-18, and TNF-α caused by SCI. Taken together, we drew a conclusion that paeonol may inhibit the activation of NLRP3 inflammasomes and pyroptosis to alleviate SCI in a rat model.

Microglial cells is commonly used as an in vitro model of CNS injury [48]. After undergoing SCI, microglial cells may be activated to secrete inflammatory cytokines and undergo changes in morphology [49]. It has been confirmed that M1 polarization of microglia is strongly correlated with the enhancement of inflammation and the damage to neuron structures, whereas M2 polarization is helpful for the repair of neurons [43]. We then detected the role of paeonol on M2 polarization of microglia, and discovered that paeonol promoted M2 polarization and inhibited M1 polarization of BV-2 cells. At the same time, we also found that the inflammation were suppressed after paeonol treatment. Our results lend credence to the previous studies [43, 50], suggesting that paeonol may promote the M2 polarization of microglial cells, thereby repressing the release of inflammatory cytokines and contributing to relieve SCI. Additionally, it is well known that microglia and astrocyte are most affected by the reduction of inflammatory response [45]. All the results suggested that paeonol may suppress the activation of BV-2 cells, promote M2 polarization, and repress the pyroptosis and formation of NLRP3 inflammasomes in vitro.

TLR4/MyD88/NF-κB (p65) signalling pathway is a primary pathway involved in inflammation of microglial cells, and the activation of this pathway is closely correlated with the expansion of inflammatory reactions [37, 38]. More importantly, the activation of TLR4/MyD88/NF-κB pathway and the up-regulation of the expression of related inflammatory factors are confirmed to aggravate SCI [51] Therefore, we studied the progression of TLR4/MyD88/NF-κB pathway in SCI, and found that paeonol reversed the promoting effects of LPS/ATP treatment on the protein levels of TLR4, MyD88, and p-p65/p65 in BV-2 cells. Similar to our findings, a study focused on the effect of paeonol on acute lung injury (ALI) has demonstrated that paeonol ameliorates LPS-induced ALI via inhibition of the TLR4/MyD88/NF-κB signalling pathway [52]. Therefore, we believed that paeonol may inactivate the TLR4/MyD88/NF-κB signalling pathway, thereby restraining the development of inflammation after SCI.

There are also some limitations of this study. First, we selected time points and concentrations based on previous literature. The therapeutic effects of paeonol on other time points remain to be explored, and it remains to be determined whether the treatment has dose dependence. Second, there are also several factors such as vascular injury, membrane/ionic dysregulation, and neurotransmitter toxicity involved in SCI progression, but this study focused on the effects of paeonol on NLRP3 inflammasomes and pyroptosis. We will elucidate these issues in future studies.

## Conclusion

In summary, our findings to some extent indicated that in the progression of SCI, paeonol may inhibit NLRP3 inflammasomes and pyroptosis through promoting M2 polarization of BV-2 cells via the TLR4/MyD88/NF-κB signalling pathway.

## Methods

### SCI rat model

Forty-eight female Sprague Dawley (SD) wild-type rats (8 weeks, 200–250 g; EseBio, Shanghai, China) were assigned randomly into four groups: the sham, SCI, SCI + carboxymethyl cellulose (CMC)-Na, and SCI + paeonol groups (n = 12). SCI rat model was established in accordance with the previous study [53]. Briefly, the rats were anaesthetized by intraperitoneal injection of pentobarbital sodium (50 mg/kg). The T9 lamina was removed after spinal exposure, followed by squeezing the spinal cord for 1 min using vascular clip (30 g forces). For the rats in the sham group, same surgical procedures were underwent except for damage to the spinal cord. Manual urinary (twice/day) was needed until the return of bladder function. Paeonol was dissolved in 0.5% CMC-Na. Subsequently, the rats in the SCI + CMC-Na and SCI + paeonol groups were intraperitoneally injected with 0.5% CMC-Na and paeonol (60 mg/kg) respectively until the rats were sacrificed. This study is reported in accordance with Animal Research: Reporting of In Vivo Experiments (ARRIVE) guidelines. All animal experiments in this study were in strict accordance with the protocols stated in the Guide for the Care and Use of Laboratory Animals and approval by ethical committee of Central Hospital Affiliated to Shandong First Medical University.

### Locomotion recovery assessment

Basso Beattie Bresnahan (BBB) scores [54] were used to assess the locomotion function of rat after SCI at the time point of 1, 3, 7, 14, and 21 days. The range of BBB scores was from 0 to 21 points. In brief, 0 points represents complete paralysis, while 21 points was on behalf of normal locomotion function. Three trained examiners who were blinded to the experimental conditions independently performed the tests to obtain the scores.

### Hematoxylin–eosin (H&E) staining assay

Seven days after SCI model establishment, the rats (n = 5) in the aforementioned four groups were euthanized by overdose of pentobarbital sodium (200 mg/kg). The spinal cord tissues (1 cm on each side of the lesion) were fixed in 4% paraformaldehyde for one day, followed by embedding in paraffin sectioned at 5 μm thickness. The sections were stained with H&E staining immediately and then were observed by a light microscopy (BX53, Olympus, Japan; magnification × 400).

### Immunohistochemistry (IHC) analysis

IHC staining was conducted using streptavidin–biotin-peroxidase complex method. Briefly, spinal cord samples were fixed, paraffin-embedded, dewaxed, rehydrated, and antigen retrieval. Then samples were stained with primary antibody anti-glial fibrillary acidic protein (GFAP) (1: 1,500; Abcam, Cambridge, MA, USA) at 4˚C overnight, followed by incubation with the secondary antibody (1:3000; Abcam) for 30 min at 37 °C. Pictures were taken under a light microscope (magnification × 400).

### Immunofluorescence labeling assay

After antigen retrieval, the samples were incubated overnight with primary antibody anti-Iba1 (1:500, Abcam) and then incubated with secondary antibody. Images were obtained with the fluorescence microscope (Olympus, Tokyo, Japan; magnification × 400). Cell counts and analysis were through ImageJ software (1.4, NIH).

### Terminal deoxynucleotidyl transferase (TdT) dUTP Nick-End Labeling (TUNEL) assay

TUNEL staining was performed using an In Situ Cell Death Detection kit (Roche, Basel, Switzerland) according to the manufacturer’s instructions. Briefly, after deparaffinization, slides were incubated with proteinase K, then TUNEL reaction mixture, followed by blocking buffer with peroxidase-streptavidin conjugate solution, and finally 0.03% diaminobenzidine. Subsequently, nuclear staining was performed with DAPI. Images were examined by a fluorescence microscope.

### Assessment for spinal cord water content

The fresh spinal cord tissues (2 mm) were initially weighted as wet weight. Afterwards, spinal cord tissues were dried at 60 °C for 72 h and the dry weight was then determined. The water content of spinal cord tissues was calculated as follows: [(wet weight—dry weight)/wet weight] × 100.

### Cell culture, grouping, and treatments

Mouse primary microglial cells (BV-2 cells) were procured from Cobioer biotech (Nanjing, China) and cultured in Dulbecco's modified Eagle's medium (DMEM) with 10% fetal bovine serum and 1% streptomycin/penicillin at 37 °C with 5% CO_2_. The cells were divided into three groups: the control, LPS/ATP, and LPS/ATP + paeonol (pae) groups. To induce NLRP3 inflammasomes, 100 ng/ml LPS was added for 24 h, and then 1 mM ATP was added for 3 h, while the cells in the control group were without any treatment. For the LPS/ATP + pae group, paeonol (15 μM) was prior to treat BV-2 cells for 1 h.

### Quantitative reverse-transcription PCR (qRT-PCR)

Total RNA was extracted from rat spinal cord tissues and BV-2 cells by Total RNA Extraction Kit (Promega, Madison, WI, USA), followed by synthesizing to cDNA using First-Strand cDNA Synthesis Kit (Thermo Fisher Scientific, Waltham, MA, USA) and performing qRT-PCR with SYBR Green FAST Mastermix (Qiagen, Dusseldorf, Germany). The expression levels were quantified by a 2^−ΔΔCt^ method. The expression of caspase 1 and NLRP3 was normalized to GAPDH, and the expression of iNOS, TNF-α, Arg-1, and IL-10 was normalized to β-actin.

### Detection of inflammatory cytokines and oxidative stress factors

Seven days after paeonol administration, inflammatory cytokines and oxidative stress factors were measured as previously described [45, 55]. In brief, spinal cord samples or BV-2 cells were homogenized in phosphate-buffered saline (PBS), subsequently centrifuged at 10,000×*g* at 4 °C for 10 min. The levels of TNF-α, IL-1β, IL-18, MDA, and GSH in the supernatant were measured using specific ELISA kits (Esebio, Shanghai, China) according to the manufacturer’s protocol.

### Western blotting analysis

RIPA buffer containing protease inhibitors was used to extract proteins from rat spinal cord tissues and BV-2 cells. Protein concentrations were then determined using a BCA Protein Assay Kit (Abcam). Protein samples (20 μg/lane) were separated via 10% SDS-PAGE and the resolved proteins were transferred onto PVDF membranes. Membranes were blocked with 5% bovine serum albumin at room temperature. After blocking, membranes were incubated overnight at 4 °C with primary antibodies against TLR4 (1:1000; Abcam), MyD88 (1:1000; Abcam), p65 (NF-κB) (1:1000; Abcam), p-p65 (phospho-NF-κB) (1:1000; Cell Signaling), NLRP3 (1:1000; Abcam), ASC (1:1000; Affinity Biosciences), caspase 1 (1:1000; Affinity Biosciences), N-GSDMD (1:1000; Abcam), and GAPDH (1:1000; Abcam). Thereafter, they were washed three times with Tris-buffered saline Tween-20. Subsequently, an HRP-conjugated IgG secondary antibody (1:5000; Santa Cruz, Waltham, MA, USA) was added and membranes were incubated at room temperature for 1 h. GAPDH was used as the internal reference. An enhanced chemiluminescence detection kit (Thermo Fisher Scientific) was used to detect the bands, which were then quantified using Gel-Pro Analyzer software (version 4.0; Media Cybernetics, Silver Spring, MD, USA).

### Statistical analysis

Data were presented as means ± SD. SPSS 23.0 software was used to perform statistical analyses. All the experiments were performed in three independent trails. Student's t-test and one-way ANOVA followed by Tukey's multiple comparisons test were used to perform the comparisons in this study. Significant difference was considered when *P* < 0.05.

## Data Availability

The data that support the findings of this study are available from Central Hospital Affiliated to Shandong First Medical University, but restrictions apply to the availability of these data, which were used under license for the current study, and so are not publicly available. Data are however available from the authors upon reasonable request and with permission of Central Hospital Affiliated to Shandong First Medical University.
